# Germ-line exon 21 EGFR V831H mutation in advanced NSCLC resistance to almonertinib: a case report

**DOI:** 10.3389/fonc.2026.1758636

**Published:** 2026-02-18

**Authors:** Daxia Cai, Jian Lou, Yanyan Zhu, Yonghui Wang

**Affiliations:** 1 Cancer Center, Lishui Central Hospital, The Fifth Affiliated Hospital of Wenzhou Medical University, Lishui Hospital of Zhejiang University, Lishui, China; 2 Department of Pharmacy, Lishui Municipal Central Hospital, The Fifth Affiliated Hospital of Wenzhou Medical University, Lishui, China

**Keywords:** almonertinib, case report, EGFR V831H, NSCLC, resistance

## Abstract

**Background:**

Germ-line EGFR mutations are rare, and their clinical significance, particularly regarding response to tyrosine kinase inhibitors (TKIs), remains poorly defined. The EGFR V831H (also known as R831H) mutation is an exceptionally rare variant with constitutive activity, and data on its therapeutic sensitivity are scarce.

**Methods:**

We present a detailed case report of a patient with advanced non-small cell lung cancer (NSCLC) harboring a germ-line EGFR V831H mutation. Diagnosis involved imaging, histopathology, and comprehensive genomic profiling of tumor tissue. Germ-line origin was confirmed via Sanger sequencing of normal patient tissue and a familial sample.

**Case presentation:**

A 68-year-old man was diagnosed with stage IIIB lung adenocarcinoma and concurrent latent tuberculosis infection (LTBI). Next-generation sequencing of a lymph node biopsy revealed co-occurring somatic KRAS G12V and an EGFR exon 21 V831H mutation, which was subsequently identified as a germ-line variant. The patient initiated antituberculosis therapy (rifampicin and isoniazid) followed by the third-generation EGFR-TKI almonertinib (110 mg/day).

**Results:**

The disease demonstrated primary resistance to almonertinib, with radiological progression in thoracic lymph nodes observed within 20 days of treatment initiation. The patient died one month later with evidence of new brain metastases.

**Conclusion:**

This case highlights primary resistance to the third-generation EGFR-TKI almonertinib in a patient with NSCLC harboring a germ-line EGFR V831H mutation. The rapid progression suggests that this specific germ-line variant may confer inherent TKI resistance, potentially exacerbated by the presence of a concurrent KRAS G12V mutation and drug-drug interactions between almonertinib and antituberculosis medications. It underscores the clinical challenge of germ-line EGFR mutations and emphasizes the need for further research to establish effective therapeutic strategies for such rare genotypes.

## Introduction

Lung cancer is responsible for the majority of cancer-related deaths globally, with a 5-year survival rate ranging from 10% to 20% (1). Non-small cell lung cancer (NSCLC) accounts for approximately 80-85% of all lung cancer cases (2). The primary risk factor for the development of lung cancer remains tobacco smoking (2). However, additional risk factors such as infectious diseases, occupational hazards, radon exposure, and genetic susceptibility are increasingly being recognized in individuals who have never smoked (3). The role of genetic factors in lung cancer was first described in 1963 (4). This observation has led to numerous studies analyzing the impact of family history on the development of lung cancer. A prospective Nordic twin study estimated that the heritability of lung cancer is approximately 18% (5). While specific germline mutations have been associated with various malignancies, their connection to lung cancer has only recently gained prominence (6). To date, the epidermal growth factor receptor(EGFR) T790M mutation has been identified as the most common germline variant; however (7–9), evidence is emerging for germline mutations in other EGFR variants as well as other oncogenes and tumor suppressor genes (7–9). In this case report, we present a novel EGFR germline mutation V831H, which showed resistance to third generation tyrosine kinase inhibitor(TKI)inhibitor.

## Case presentation

**Figure 1:**
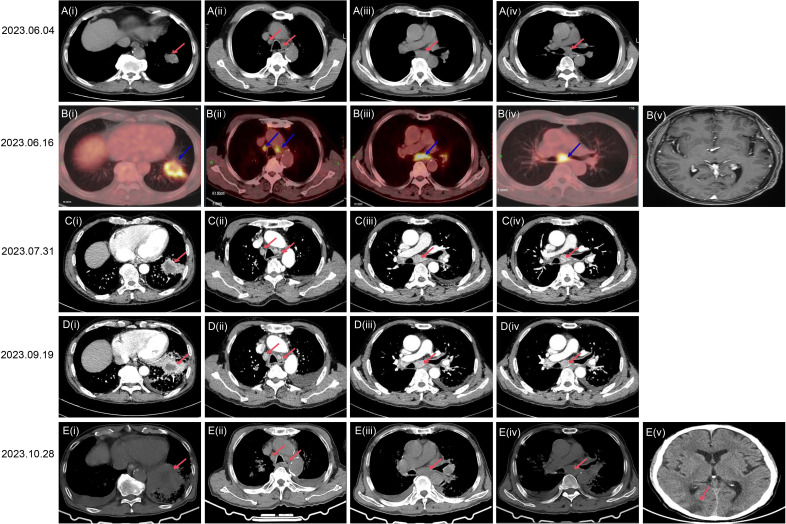
CT, PET and MRI scan images showing various scans of the chest and brain during different treatment time.

**Figure 2:**
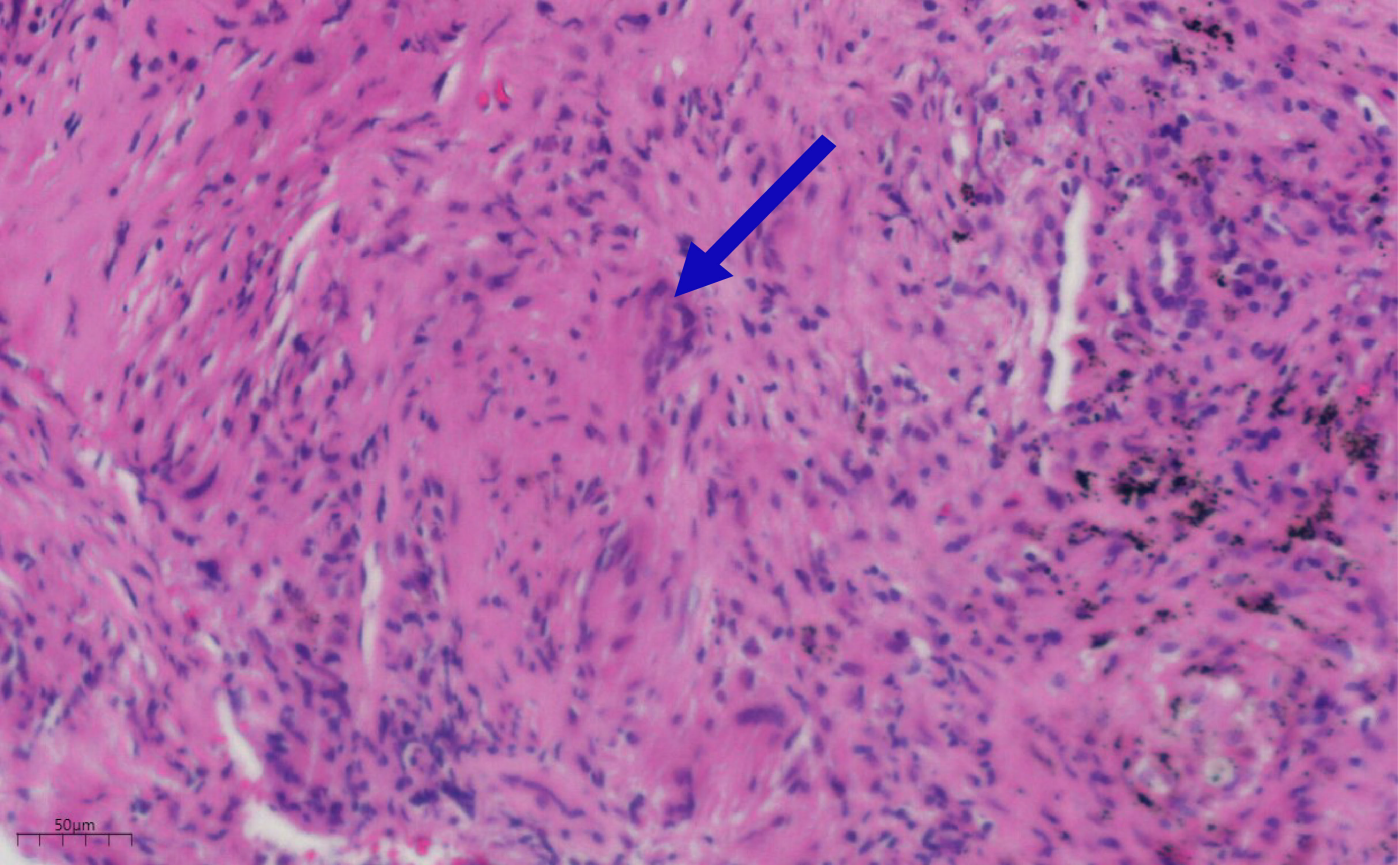
Pathological examination of the lung lesion biopsy specimens under CT guidance revealed the presence of granulomatous lesions.

The patient is a 68-year-old male. On June 4, 2023, during a routine physical examination at the local hospital, a computed tomography (CT) scan revealed a space-occupying lesion in the lateral basal segment of the left lower lung. The size of the lesion was approximately 45 × 31 millimeters(mm). The mass contained calcification and air-filled spaces (bubbles), had a lobulated edge, had slender needle-like structures, and showed local pleural traction. Clearly enlarged lymph nodes were visible in the mediastinum. No pleural thickening was observed, and no significant free pleural effusion was found in the thoracic cavity (**Figure 1A**). Subsequently, on June 16, 2023, a positron emission tomography-computed tomography (PET-CT) performed at our hospital confirmed an irregularly shaped mass in the left lower lung. It was an irregular mass shadow in the left lower lobe, measuring approximately 39×29 mm with heterogeneous density. Increased uptake was notably observed in the surrounding area (maximum SUV: 6.9), accompanied by distinct defect changes within the central portion. The internal bronchus appears partially obstructed or narrowed, while multiple patchy ground-glass opacities were present in the distal lung tissue (**Figure 1B**(i). Multiple enlarged lymph nodes were observed within the mediastinum and bilateral hilar regions. The largest node resided in the subcarinal lymph nodes, measuring about 1.4 cm in short diameter, demonstrating increased uptake and reaching a maximum SUV of 8.1 (**Figure 1B**(ii-iv). Brain magnetic resonance imaging (MRI) examination showed no abnormalities (**Figure 1B**(v). The patient did not receive further treatment due to personal family matters requiring attention. One and a half months later (July 31, 2023), a CT scan showed an irregular mass in the left lower lobe, approximately 52×27 mm in size (visible on mediastinal window), with heterogeneous density and partial obstruction of the internal bronchus.Several patchy blurred shadows were observed in the surrounding lung tissue; the lesion exhibited significant heterogeneous enhancement with patchy hypo-enhanced necrotic areas inside (**Figure 1C**(i). The peripheral obstructive inflammation has progressed, with a small amount of left pleural effusion, the mediastinal and bilateral hilar lymph nodes have enlarged compared to before, indicating disease progression (**Figure 1C**(i-iv). Brain MRI, abdominal CT, and supraclavicular lymph node B-mode ultrasound examination showed no abnormalities. Although the results of acid-fast staining, periodic acid-Schiff staining and periodic acid-silver staining were all negative, the pathological examination of the lung lesion biopsy specimens under CT guidance revealed the presence of granulomatous lesions (**Figure 2**). Subsequently, the peripheral blood T-SPOT (T-cell Spot of IFN-γ Release Assay) test was positive; bronchoscopy lavage fluid examination indicated a weakly positive TBNDA. On August 7, 2023, the patient was diagnosed with latent tuberculosis infection (LTBI) after MDT (Multidisciplinary Team) discussion. Further examination via transbronchial needle aspiration (TBNA) of the subcarinal lymph nodes revealed the following immunohistochemical features: AE1/AE3 (+), TTF-1 (+), NapsinA (+), CK7 (+), P40 (-), CK5/6 (-), and Ki-67 (25%+), leading to a diagnosis of non-small cell lung cancer (NSCLC). Additionally, genetic testing was further completed. Additionally, the tumor proportion score (TPS) for PD-L1 expression detected using the anti-PD-L1 antibody clone E1L3N was < 1%. Therefore, the patient was diagnosed with stage IIIB (T3N2M0) LUAD(lung adenocarcinoma) and LTBI. The patient received anti-tuberculosis treatment with rifampicin and isoniazid.

**Figure 3:**
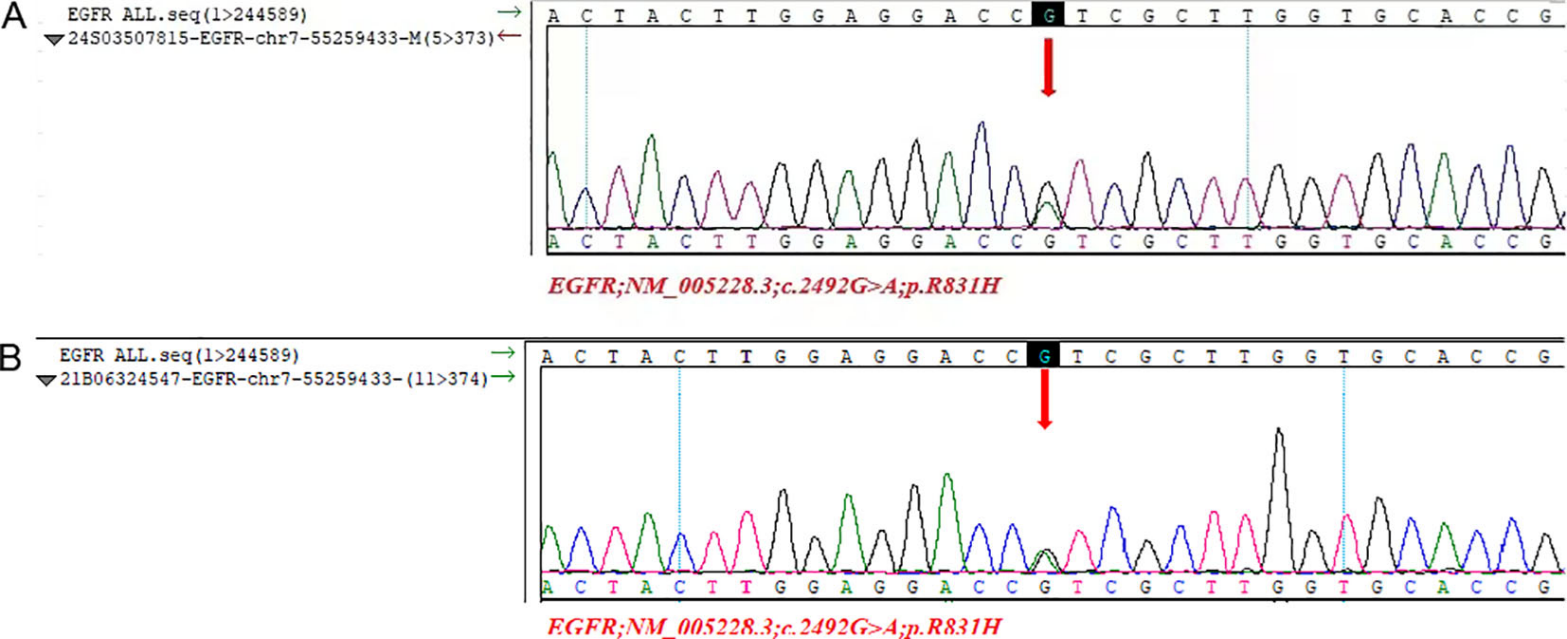
Representative electropherogram for EGFR sequencing of the samples of the patient and his son. **(A)** DNA sequencing electrophoretograms for DNA obtained from normal tissue of the patient, identifying one EGFR exon 21 mutation, the V831H variant, is present and confirming it is germ-line; **(B)** DNA sequencing electrophoretograms for DNA obtained from white blood of the patient son, identifying one germ-line EGFR exon 21 mutation.

Bronchial Artery Embolization (BAE) is an interventional radiological procedure that involves using a catheter to block the bronchial arteries, thereby reducing the blood flow to the bleeding site and controlling the symptoms of hemoptysis (coughing up blood). This is a standard palliative treatment for patients with severe or recurrent hemoptysis due to lung diseases (such as bronchiectasis, lung cancer, or tuberculosis, etc.). For lung cancer patients with chemotherapy contraindications, BAE is a good treatment option. Given the presence of LTBI and the 15-day turnaround time for genetic testing results, BAE treatment was initiated on August 9, 2023. On August 24, 2023, next-generation sequencing (NGS)-based whole-genome analysis of hilar lymph nodes detected EGFR exon 21 V831H and KRAS G12V mutations. The patient received oral administration of 110mg (milligram) Almonertinib Capsules once daily as targeted therapy on August 25, 2023. However, on September 19, 2023, a CT scan showed an irregular mass in the left lower lobe, approximately 55×36 mm in size (visible on mediastinal window), with flocculent low-density areas inside; the internal bronchus had partial stenosis and obstruction, and the surrounding lung tissue showed multiple patchy ground-glass opacities and reticular changes, while the solid components exhibited moderate enhancement on contrast-enhanced scan (**Figure 1D**(i). There was enlargement of mediastinal and hilar lymph nodes, obstructive pneumonia in the left lower lobe complicated with carcinomatous lymphangitis, and an increased left pleural effusion, indicating disease progression (**Figure 1D**). On October 28, 2023, a Chest CT showed an irregular mass in the left lower lobe, approximately 71×9 mm in size, with low-density interior; there were partial bronchial stenosis and obstruction, accompanied by multiple patchy ground-glass opacities and reticular changes in the surrounding lung tissue (**Figure 1E**(i). Enlargement of the mediastinal and hilar lymph nodes had increased compared to before (**Figure 1E**(ii-iv). And the patient passed away due to brain metastases on October 28th, 2023 (**Figure 1E**(v). EGFR V831H mutation was also detected in the patient's normal lung tissue and his son's leukocytes via sanger sequencing, confirming it as a germline mutation. The representative electropherogram for EGFR sequencing of the samples of the patient and his son was shown in **Figure 3**. And a clear and structured timeline for the diagnostic procedures and treatment steps of the case was shown in **Table 1**.

**Table 1:** A clear and structured timeline for the diagnostic procedures and treatment steps of the case.

Date	Diagnostic procedures/treatment
2023.6.4	A CT scan performed at the local hospital detected a mass in the right lower lung along with mediastinal lymphadenopathy.
2023.6.16	PET/CT scan demonstrated lung cancer accompanied by mediastinal and pulmonary hilum lymph node metastasis.
2023.7.31	The patient successfully completed a contrast-enhanced CT examination.
2023.8.1	The patient underwent a CT-guided percutaneous biopsy of a mass located in the right lower lung.
2023.8.2	The pathological results of the biopsy revealed granulomatous lesions.
2023.8.4	The patient underwent a puncture biopsy of the mediastinal lymph nodes under fiberoptic bronchoscopy.
2023.8.7	After a multidisciplinary discussion, the patient was diagnosed with stage IIIB (T3N2M0) non-small cell lung cancer (NSCLC) and latent tuberculosis infection (LTBI), and anti-tuberculosis treatment and BAE (Bronchial Artery Embolization) therapy should be initiated.
2023.8.9	BAE treatment was administered for the patient. Commence standard isoniazid and rifampin combination therapy for anti-tuberculosis treatment.
2023.8.24	NGS(next-generation sequencing) genetic testing report on subcarinal adenocarcinoma tissue: EGFR V831H mutation positive
2023.8.25	The patient underwent oral targeted therapy using the third-generation EGFR-TKI(amelitinib).
2023.9.19	Follow-up enhanced CT demonstrated progression of the right lower lung tumor.
2023.10.28	The patient succumbed to metastatic brain cancer originating from lung cancer.

## Discussion

Studies have found that germline mutations are mainly associated with lung adenocarcinoma (LUAD), but they can also occur in other subtypes such as adenosquamous carcinoma (ASC), although they are much rarer in the latter (10, 11). In advanced NSCLC, germline mutations often coexist with somatic mutations, for example, germline T790M mutations are frequently accompanied by somatic L858R mutations, which may influence tumorigenesis and progression (12–14). As a third-generation EGFR-TKI, Osimertinib is recommended for the treatment of patients with T790M mutations. A case report showed that a patient with stage IV LUAD carrying a germline EGFR T790M mutation achieved a 19-month progression-free survival (PFS) after treatment with Osimertinib, indicating its certain efficacy against germline mutations (15). Additionally, Osimertinib is a standard treatment option, especially in cases of acquired or primary resistance (10, 16, 17). Germline mutation-induced lung cancer may exhibit intrinsic resistance to traditional EGFR-TKIs (e.g., first- and second-generation agents), as the T790M mutation itself is a resistance mechanism (18–20). However, studies have shown that personalized therapies designed for germline mutations (such as combining molecular targeted drugs) can improve prognosis (11, 21). For example, in patients without other driver mutations, EGFR-TKIs may still show some sensitivity, but more data are needed to support this (11, 22). In clinical trials, compounds targeting the T790M mutation (e.g., MG-3C) have demonstrated the ability to selectively kill lung cancer cells carrying this mutation, but their application in germline mutations is still in the exploratory stage (23). Additionally, treatment regimens for rare mutations such as R776H and V843I are still under investigation, with no clear guidelines available (24, 25). Studies have also pointed out that germline mutations may lead to innate resistance to EGFR-TKIs, which requires in-depth research into tumor heterogeneity and clonal evolution mechanisms (18, 26, 27).

Germline EGFR mutations are extremely rare in NSCLC, accounting for only a small fraction of all cases. For example, a large-scale study analyzed 31,906 lung cancer patients and found only 64 cases with germline variants, of which 22 were germline EGFR mutations, accounting for merely 0.2% of the total cases (28). In another retrospective analysis of 12,833 Chinese lung cancer patients, the overall detection rate of germline EGFR and ERBB2 mutations was also very low (11). Specifically, the germline T790M mutation (a common type of EGFR mutation) has an incidence of approximately 1% in NSCLC, highlighting its rarity in the population (10, 14). Additionally, in a study conducted in Azerbaijan, genetic testing was performed on 507 NSCLC patients, and only 11 cases (2.1%) carried the germline T790M variant, with most of these patients having a family history of lung cancer[13]. Germline EGFR mutations mainly include T790M, R776H, V843I, P848L, and K757R, among which T790M is the most common type. For instance, a study summarized common germline mutation sites, including T790M, V843I, R776H, and P848L, which are usually identified in lung cancer family lineages (29, 30). Studies have found that among 22 patients with germline EGFR mutations, 95.5% had the T790M variant, and 50% also had concurrent L858R somatic mutations (i.e., acquired mutations within tumor cells) (31). Additionally, studies on Chinese patients have identified EGFR K757R as another relatively common but highly diverse germline mutation (11). These mutation types play a key pathogenic role in familial lung cancer. For example, a case report described three sisters and their mother all carrying the rare germline EGFR p.R776H mutation, emphasizing genetic susceptibility (22, 24). Germline mutations are strongly associated with familial lung cancer. In the study of 22 patients, 59.1% had a family history of lung cancer, and all 11 germline T790M carriers in the Azerbaijani cohort had a family history (12, 13, 31). A case report showed that germline T790M mutations can cause disease in multiple generations of family members, such as a mother and son being diagnosed with advanced metastatic LUAD simultaneously (32).

This report describes a case of NSCLC involving germline EGFR mutations. It is worth noting that aematinib, as a targeted therapeutic drug for EGFR mutation R831H, has shown poor efficacy. Germ-line EGFR variants have been rarely described, and the EGFR mutation R831H belongs to a group of very rare EGFR mutations with constitutive characteristics that are not always demonstrated. Only two studies found 2 and 4 germ-line EGFR mutation R831H cases among 12,833 and 31,906 lung cancer cases, respectively (7, 8). Due to its rarity, no therapeutic strategy has been established to address germ-line mutations in EGFR R831H. To the best of our knowledge, only two other cases of germ-line mutations in EGFR R831H with treatment response have been documented, including one case of erlotinib monotherapy and one case of gefitinib treatment (7). Both cases responded to TKI inhibitors. In this case, the patient did not respond to almonertinib, a third-generation EGFR inhibitor. Germ-line EGFR R831H are rare but may contribute to oncogenesis. In prostate cancer, tumor tissue or cell line with germ-line EGFR R831H mutation showed increased phosphorylation level, activation of downstream pathway and increased migration ability, indicated constitutive activity of EGFR, which were the main causes of tumorigenesis. This maybe indicated that high level target drug needed to be used. Benesova et al. found that patients with concurrent EGFR and KRAS mutations had an initial positive response to EGFR-TKI treatment but not last long, just 3 to 6 months (33), indicating concurrent EGFR and KRAS mutation lead to poor response.

As an extremely rare gene mutant located in exon 21 of the EGFR kinase domain, V831H exhibits unique biological properties, which may account for its resistance to third-generation TKIs (e.g., almonertinib). The underlying mechanisms may involve compound mutations, structural alterations, signaling pathway dysregulation, and epigenetic modifications. Consistent with the "two-hit hypothesis" (34) regarding tumorigenesis induced by mutant EGFR, EGFR V831H alone is insufficient to trigger carcinogenesis and requires synergy with other somatic mutations—for example, the KRAS G12V mutation in our patient likely activated alternative oncogenic pathways, thereby counteracting the efficacy of TKIs when EGFR and KRAS mutations coexist. Secondary EGFR mutations (T790M, C797S) (35–37), which are known to drive resistance to first-/second-generation and third-generation TKIs respectively, further highlight the genetic complexity of V831H-related resistance, although these mutations were not detected in this case. Structural analysis indicates that the valine-to-histidine substitution at the V831 site disrupts the conformational stability of the activation loop in the EGFR kinase domain, thereby reducing the binding affinity of third-generation tyrosine kinase inhibitors while preserving kinase activity—a pattern consistent with rare EGFR mutations such as V834L (38). Preclinical data from prostate cancer models also indicate that V831H leads to sustained activation of the downstream PI3K-AKT and RAS-MAPK pathways, which is achieved by increasing EGFR phosphorylation. This allows tumor cells to proliferate without being inhibited by EGFR-TKIs. Additionally, emerging evidence suggests that germline variants such as USP36 rs3744797 may regulate TKI sensitivity through m6A methylation epigenetic modifications—a direction that warrants further research on V831H (39, 40,. Clinically, limited but valuable data on V831H provide key insights for treatment optimization: Two previous cases of non-small cell lung cancer (NSCLC) with V831H mutations achieved partial responses with erlotinib (8 months) and gefitinib (6 months) respectively (31, 32). Studies have shown that in cases with isolated V831H mutations, the activity of first-generation tyrosine kinase inhibitors (TKIs) is significantly higher than that of third-generation agents such as almonertinib—especially in the presence of co-occurring mutations or comorbidities (e.g., latent tuberculosis infection in our patient). The antagonistic interaction between almonertinib and anti-tuberculosis drugs (rifampicin and isoniazid, potent CYP3A4 inducers) further reduces drug concentrations, highlighting the need to avoid such drug interactions or adjust treatment regimens (33). For V831H-mutated patients with TKI resistance, comprehensive genomic analysis to detect concurrent somatic mutations, PD-L1 expression testing (our patient's TPS < 1% limits the use of immune checkpoint inhibitors), and consideration of combination therapies (e.g., EGFR-TKI + MEK inhibitors for KRAS co-mutations) are critical for guiding personalized treatment (34, 35). The patient harbored both germline EGFR V831H and somatic KRAS G12V mutations. Our case suggested that KRAS G12V activated alternative oncogenic pathways, counteracting TKI efficacy—a mechanism not reported in prior germline R831H cases. Sanger sequencing detected EGFR V831H in the patient’s normal lung tissue and his son’s leukocytes, confirming it as a germline mutation. This is the first report of germline V831H with documented inheritance, whereas prior R831H cases did not mention familial transmission. Despite this, our study, like research on rare gene mutations, has several limitations: the sample size of V831H mutation cases is small, and there is a lack of structural and functional experiments to clarify how V831H synergizes with mutations such as KRAS G12V to drive tumorigenesis and TKI resistance. Future studies should employ methods including molecular modeling, cell line transfection, and kinase activity assays to define the impact of V831H on EGFR structure; investigate how germline EGFR mutations regulate the tumor microenvironment and immune evasion (via PD-L1 upregulation or recruitment of immunosuppressive cells); and evaluate targeted combination strategies, such as inhibitors of EGFR nuclear translocation or epigenetic modulators, to overcome resistance caused by nuclear EGFR or m6A modifications.

The patient was LTBI, which is also the reason for his poor treatment response. Studies showed that male lung cancer patients with a history of tuberculosis have poor clinical outcomes with EGFR-TKIs (41), and compared with patients without a history of TB infection, patients with TB infection who received TKI for NSCLC had poorer PFS(Progression-Free Survival) and OS(Overall Survival) (42). Almonertinib is a novel third-generation EGFR tyrosine kinase inhibitor (TKI) primarily metabolized by the CYP3A enzyme. In vitro studies have shown that after metabolism by CYP3A, almonertinib is mainly converted into the N-demethylated metabolite HAS-719, which is the primary active component in human plasma (43). Pharmacokinetic studies in healthy volunteers demonstrated significant changes in the pharmacokinetic parameters of almonertinib when co-administered with CYP3A inhibitors (e.g., itraconazole) or inducers (e.g., rifampicin). Specifically, rifampicin—a potent CYP3A inducer—reduced the peak plasma concentration (Cmax) and area under the curve (AUC) of almonertinib by 79.3% and 92.6%, respectively, while unexpectedly decreasing the AUC of HAS-719 by 72.5%[43]. In addition, in vitro experiments confirmed that both almonertinib and HAS-719 are substrates of CYP3A and P-glycoprotein (P-gp). In a beagle dog model, co-administration with rifampicin reduced the fecal recovery of almonertinib and HAS-719 and significantly increased the levels of further metabolites of HAS-719, indicating that rifampicin-induced enhancement of CYP3A activity promotes the further metabolism of HAS-719 rather than altering excretion (43). In human pharmacokinetic evaluations, the exposure of a single dose of almonertinib (110 mg) was significantly reduced when co-administered with rifampicin, confirming that almonertinib is a moderately sensitive substrate of CYP3A in vivo (44). Therefore, the pharmacokinetics of almonertinib are susceptible to the effects of CYP3A modulators, and special attention should be paid to drug-drug interactions in clinical practice (43). Rifampicin, a potent CYP3A4 inducer, significantly reduces the exposure levels of third-generation EGFR tyrosine kinase inhibitors (TKIs) such as osimertinib. In clinical studies of osimertinib, co-administration with rifampicin led to a 27% reduction in peak plasma concentration (Cmax) and a 22% reduction in area under the curve (AUC) of osimertinib, respectively. These changes were below the predefined non-inferiority threshold (lower limit of 50%), indicating that the inductive effect of rifampicin becomes apparent within 7 days of initiation (45). Additionally, after discontinuation of rifampicin, the exposure levels of osimertinib required 3 weeks to return to baseline (45). Similarly, in another study, rifampicin—acting as a potent CYP3A4 inducer—significantly reduced the exposure of afatinib (alflutinib) and its active metabolite AST5902, resulting in a 39% decrease in AUC and a 42% decrease in Cmax, respectively. The study recommended avoiding co-administration of potent CYP3A4 inducers during afatinib treatment[46]. These findings are consistent with data on almonertinib, where rifampicin also substantially reduced the exposure of almonertinib and its metabolite HAS-719 (43). Thus, rifampicin reduces the systemic exposure of third-generation TKIs by inducing CYP3A4, which may compromise the efficacy of almonertinib (43, 45, 46).

Therapeutic Drug Monitoring (TDM) has been considered and applied in the management of tyrosine kinase inhibitors (TKIs), including third-generation EGFR-TKIs, to optimize dosing and reduce toxicity. TDM helps address the significant pharmacokinetic variability of TKIs, such as their narrow therapeutic window and the correlation between exposure levels and efficacy/toxicity (47). Studies have shown that TDM has potential in patients with EGFR-mutated lung cancer for individualized dose adjustment (48). TDM has been applied to various TKIs, including sorafenib, imatinib, sunitinib, and osimertinib, to monitor plasma drug concentrations and predict efficacy and tolerability (49–52). In sunitinib treatment for metastatic renal cell carcinoma, TDM was evaluated as a feasible tool to manage highly variable exposure (51). In non-small cell lung cancer (NSCLC), TDM has been proposed for EGFR-TKIs (including second- and third-generation inhibitors) to improve safety and efficacy, especially when active metabolites are present (e.g., metabolites of osimertinib), requiring simultaneous monitoring of both parent drug and metabolite concentrations (53, 54, 48). Thus, TDM serves as a clinical strategy to detect reduced exposure caused by CYP3A4 inducers such as rifampicin. Resistance to third-generation EGFR tyrosine kinase inhibitors (TKIs) such as osimertinib is a critical challenge in the treatment of EGFR-mutated non-small cell lung cancer (NSCLC). The resistance mechanisms are diverse and unpredictable, potentially leading to rapid disease progression (55, 56). After osimertinib resistance develops, patients may exhibit AXL kinase-mediated resistance mechanisms (57). Studies have found that reduced exposure (e.g., caused by CYP3A4 induction) may affect efficacy, but the rapidity of disease progression is more attributed to intrinsic tumor mechanisms, such as EGFR T790M mutation or activation of other bypass signaling pathways (55, 56). Therefore, reduced exposure may contribute to treatment failure, but it is insufficient to explain the rapidity of disease progression alone; the combined effects of resistance mechanisms are more critical[55,56]. Targeted drug resistance encompasses resistance resulting from mutations in additional genes (58). Only 50 NSCLC-related genes were detected in this patient. In order to find the cause of drug resistance in patients, adding the list of genes tested, like whole exome sequencing (WES)or whole genome sequencing(WGS), may be a powerful means.

The clinical significance of this case, as well as its potential impact on future diagnostic or therapeutic decisions, should be noted by clinicians. From this case, we should draw the following lessons:

1). Importance of Comprehensive Genetic Testing in Germline Mutation Screening. The identification of a germline EGFR V831H mutation in the patient’s normal lung tissue and his son’s leukocytes—with this result confirmed in the patient—underscores the necessity of germline testing for patients with early-onset or familial non-small cell lung cancer (NSCLC), even when the mutation is rare. Additionally, cascade testing of family members is essential to identify carriers, who may require enhanced surveillance measures such as annual low-dose computed tomography (CT) scans.
2). Complexity of Resistance in EGFR/KRAS Co-Mutated NSCLC The patient’s rapid disease progression during treatment with almonertinib, an EGFR tyrosine kinase inhibitor (TKI), despite harboring a germline EGFR mutation suggests two key implications: first, concurrent KRAS G12V mutations may confer primary resistance to EGFR TKIs even in cases with germline EGFR mutations; second, combination therapies (e.g., EGFR TKIs plus KRAS inhibitors) or immunotherapy-based strategies—despite low PD-L1 expression—may be more effective for such patients, highlighting the need for further clinical trials to validate these approaches.
3). Balancing Anti-Tuberculosis Therapy and Cancer Treatment The co-diagnosis of latent tuberculosis infection (LTBI) and NSCLC emphasizes two critical clinical needs: multidisciplinary team (MDT) collaboration to prioritize treatment sequencing, such as initiating anti-tuberculosis therapy before systemic cancer treatment to mitigate infection risk; and close monitoring of LTBI patients during cancer therapy, as immunosuppression associated with cancer treatment may reactivate latent tuberculosis.

## Data Availability

The original contributions presented in the study are included in the article/supplementary material. Further inquiries can be directed to the corresponding author.
