# Correction: Germ-line exon 21 EGFR V831H mutation in advanced NSCLC resistance to almonertinib: a case report

**DOI:** 10.3389/fonc.2026.1836262

**Published:** 2026-04-13

**Authors:** Daxia Cai, Jian Lou, Yanyan Zhu, Yonghui Wang

**Affiliations:** 1 Cancer Center, Lishui Central Hospital, The Fifth Affiliated Hospital of Wenzhou Medical University, Lishui Hospital of Zhejiang University, Lishui, China; 2 Department of Pharmacy, Lishui Municipal Central Hospital, The Fifth Affiliated Hospital of Wenzhou Medical University, Lishui, China

**Keywords:** almonertinib, case report, EGFR V831H, NSCLC, resistance

There was a mistake in **Table 1** as published.

**Table 1:** A clear and structured timeline for the diagnostic procedures and treatment steps of the case.

Date	Diagnostic procedures/treatment
2020.6.4	A CT scan performed at the local hospital detected a mass in the right lower lung along with mediastinal lymphadenopathy.
2025.7.16	PET/CT scan demonstrated lung cancer accompanied by mediastinal and pulmonary hilum lymph node metastasis.
2023.7.31	The patient successfully completed a contrast-enhanced CT examination and has been scheduled for a percutaneous lung biopsy.
2023.8.1	The patient underwent a CT-guided percutaneous biopsy of a mass located in the right lower lung.
2023.8.2	The pathological results of the biopsy revealed granulomatous lesions.
2024.8.4	The patient underwent transbronchoscopic biopsy of the subcarinal lymph nodes.
2024.8.7	Following multidisciplinary discussion, consensus was reached that the patient has latent tuberculosis and that anti-tuberculosis therapy should commence.
2024.8.9	BAE (Bronchial Artery Embolization) treatment was administered for lung Cancer. Commence standard isoniazid and rifampin combination therapy for anti-tuberculosis treatment.
2024.8.13	Pathological examination of the histological wax block via subcarinal puncture confirmed adenocarcinoma.
2023.8.19	The patient underwent bronchial angiography and embolization in an effort to control the tumor.
2023.8.24	NGS genetic testing report on subcarinal adenocarcinoma tissue: EGFR V831H mutation positive.
2023.8.25	The patient underwent oral targeted therapy using the third-generation EGFR-TKI(amelitinib).
2023.9.19	Follow-up enhanced CT demonstrated progression of the right lower lung tumor.
2023.10.28	The patient succumbed to metastatic brain cancer originating from lung cancer.

The incorrect table is as follows:

The modified parts are marked in red font. The details are as follows:

The date “2020.6.4” should be changed to “2023.6.4”;

The date “2025.7.16” should be changed to “2023.6.16”;

The sentence “The patient successfully completed a contrast-enhanced CT examination and has been scheduled for a percutaneous lung biopsy.”should be changed to “The patient successfully completed a contrast-enhanced CT examination.”;

The date “2024.8.4” should be changed to “2023.8.4”;

The sentence “The patient underwent transbronchoscopic biopsy of the subcarinal lymph nodes.”should be changed to”The patient underwent a puncture biopsy of the mediastinal lymph nodes under fiberoptic bronchoscopy.”;

The date “2024.8.7” should be changed to “2023.8.7”;

The sentence “Following multidisciplinary discussion, consensus was reached that the patient has latent tuberculosis and that anti-tuberculosis therapy should commence.”should be changed to “After a multidisciplinary discussion, the patient was diagnosed with stage IIIB (T3N2M0) non-small cell lung cancer (NSCLC) and latent tuberculosis infection (LTBI), and anti-tuberculosis treatment and BAE (Bronchial Artery Embolization) therapy should be initiated.”;

The date “2024.8.13” should be changed to “2023.8.9”;

The sentence “Pathological examination of the histological wax block via subcarinal puncture confirmed adenocarcinoma.”should be changed to “BAE treatment was administered for the patient. Commence standard isoniazid and rifampin combination therapy for anti-tuberculosis treatment.”

The date “2023.8.19” and the sentence “The patient underwent bronchial angiography and embolization in an effort to control the tumor.” should be deleted;

The sentence “NGS genetic testing report on subcarinal adenocarcinoma tissue: EGFR V831H mutation positive”should be changed to “NGS(next-generation sequencing) genetic testing report on subcarinal adenocarcinoma tissue: EGFR V831H mutation positive”.

The corrected **Table 1** appears below.

**Table 1:** A clear and structured timeline for the diagnostic procedures and treatment steps of the case.

Date	Diagnostic procedures/treatment
2023.6.4	A CT scan performed at the local hospital detected a mass in the right lower lung along with mediastinal lymphadenopathy.
2023.6.16	PET/CT scan demonstrated lung cancer accompanied by mediastinal and pulmonary hilum lymph node metastasis.
2023.7.31	The patient successfully completed a contrast-enhanced CT examination.
2023.8.1	The patient underwent a CT-guided percutaneous biopsy of a mass located in the right lower lung.
2023.8.2	The pathological results of the biopsy revealed granulomatous lesions.
2023.8.4	The patient underwent a puncture biopsy of the mediastinal lymph nodes under fiberoptic bronchoscopy.
2023.8.7	After a multidisciplinary discussion, the patient was diagnosed with stage IIIB (T3N2M0) non-small cell lung cancer (NSCLC) and latent tuberculosis infection (LTBI), and anti-tuberculosis treatment and BAE (Bronchial Artery Embolization) therapy should be initiated.
2023.8.9	BAE treatment was administered for the patient. Commence standard isoniazid and rifampin combination therapy for anti-tuberculosis treatment.
2023.8.24	NGS(next-generation sequencing) genetic testing report on subcarinal adenocarcinoma tissue: EGFR V831H mutation positive
2023.8.25	The patient underwent oral targeted therapy using the third-generation EGFR-TKI(amelitinib).
2023.9.19	Follow-up enhanced CT demonstrated progression of the right lower lung tumor.
2023.10.28	The patient succumbed to metastatic brain cancer originating from lung cancer.

The original version of this article has been updated.

